# Invasive Lobular Carcinoma of the Breast with Extracellular Mucin: Case Report of a New Variant of Lobular Carcinoma of the Breast

**DOI:** 10.1155/2018/5362951

**Published:** 2018-04-05

**Authors:** M. Boukhechba, H. Kadiri, B. El Khannoussi

**Affiliations:** Department of Pathology, National Institute of Oncology, Rabat, Morocco

## Abstract

Invasive carcinoma of no special type (NST) or ductal carcinoma is the largest group of invasive breast cancers. Invasive lobular carcinoma (ILC) is the second most common histological type; it comprises 5%–15% of all invasive breast cancers. Historically, lobular neoplasia and invasive lobular carcinoma may produce intracellular mucin that pushes the nucleus to one side, creating the characteristic signet ring cell morphology. The extracellular mucin secretion is essentially described in mucinous breast carcinoma. Mucinous differentiation can be seen in small areas of NST carcinoma, but recently a few cases of invasive lobular carcinoma with extracellular mucin are reported in the literature. It is important for pathologists to recognize this new entity because it mimics a NST carcinoma, as such a diagnosis may require a different approach in clinical management and surveillance. We report a new case of ILC with extracellular mucin and a review of the literature.

## 1. Introduction

Invasive lobular carcinoma (ILC) is a distinct subtype of breast carcinomas; the classical invasive lobular carcinoma is characterized by a bland cytology, loss of cell cohesion, and a diffuse single cell infiltration pattern. In situ lesions of lobular carcinoma have the same morphology and are found in approximately 58–98% of cases [1].

ILC has some histologic variants that differ from the classical type in terms of their histological growth or cytological patterns but still lack cellular cohesion.

Although tumor cells may contain intracytoplasmic mucin secretion and demonstrate a signet-ring appearance, extracellular mucin secretion is exceptionally seen in ILC. We herein report a rare case of ILC with extracellular mucin. To the best of our knowledge, only 13 cases have been reported in the literature [2–7].

## 2. Case History

**Figure 1:**
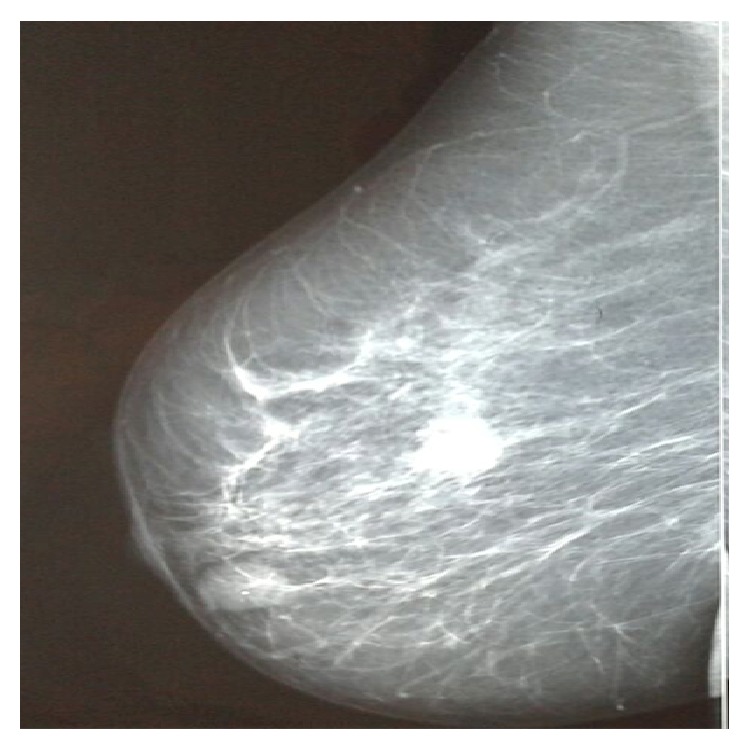
Mammography of the right breast showing spiculated, irregular mass lesion measuring 1,5 × 1,4 cm, located at upper-outer quadrant. A secondary retromammary lesion measuring 1,9 × 1 cm was also detected.

**Figure 2:**
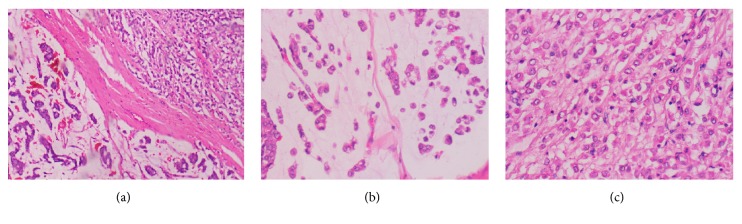
Invasive lobular carcinoma of the breast with extra cellular mucin shows the classic pattern of lobular carcinoma with single cell infiltration and discohesive pattern (top); groups of tumor cells are seen floating in a pool of extracellular mucin (bottom) (a: hematoxylin-eosin; original magnification ×20). Extracellular mucin lakes with clusters of tumor cells (b: hematoxylin-eosin; original magnification ×40). Areas of classic invasive lobular carcinoma showing typical single cell infiltration of the stroma and discohesive pattern (c: hematoxylin-eosin; original magnification ×40).

**Figure 3:**
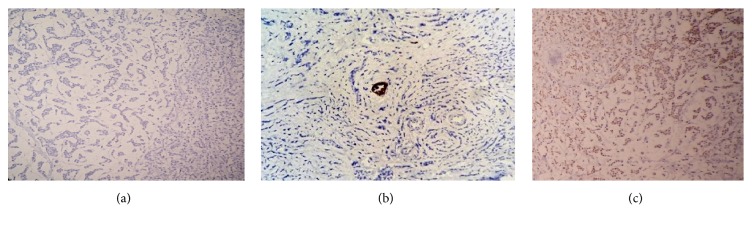
Immunohistochemical stain showed absence of membranous E-cadherin staining in the classical invasive lobular carcinoma and in the cells surrounded by extracellular mucin (a: E-cadherin ×20) with positive internal control (b: E-cadherin ×40). The tumor cells were positive for ER (c: ER ×20) and negative for PR and HER2 (images not shown).

A 75-year-old postmenopausal woman without family history of breast cancer presented with a mass in the right breast. No axillary lymphadenopathy was detected upon examination. Mammography indicated two lesions (Figure 1). There was a primary irregular lesion measuring 1,5 × 1,4 cm, located at upper-outer quadrant of the right breast, BI-RADS category was assessed to be 5, there was a second retromammary lesion measuring 1,9 × 1 cm with a benign appearance of category BI-RADS 1, core needle biopsy was performed in the primary suspect lesion, the microscope examination showed the presence of small uniform tumor cells floating in lakes of extracellular mucin, some cells showed signet ring cell morphology (Figures 2(a) and 2(b)), and areas of classical lobular carcinoma were noted with single cell infiltration (Figure 2(c)). In situ lesions of lobular or ductal carcinoma were not observed. On immunohistochemistry examination, The E-cadherin was negative in both areas of the tumor with positive internal control (Figures 3(a) and 3(b)) and the lobular origin was confirmed; chromogranin A and synaptophysin were also negative. Prognostic and predictive marker studies showed the positivity for estrogen (Figure 3(c)). Progesterone and HER2 were negative.

## 3. Discussion

Invasive lobular carcinoma (ILC) is the second most common histological type of breast carcinoma; it comprises 5%–15% of all invasive breast cancers. In comparison with invasive ductal carcinomas, it has a higher incidence of multiplicity, bilaterality, and a tendency to metastasize to particular sites such as genital tracts, retroperitoneum, and meninges [1, 2].

Grossly, it may present as mass with irregular borders that sometimes can be difficult to detect on gross examination, and the breast tissue appears normal with only a firm consistency by palpation [1].

Histologic variants of invasive lobular carcinoma are classic, solid, alveolar, pleomorphic, tubulolobular, signet ring cell, and mixed type. All have in common a loss of cellular cohesion, the classic invasive lobular carcinoma is characterized by proliferation of discohesive small cells individually dispersed or arranged with a typical single-file pattern without destruction of breast tissue, the nuclei of cells are round with little mitotic activity, the tumor cells usually present a concentric pattern around existing ducts and lobular units termed targetoid pattern, and the solid variant consists of sheets of lobular cells that have pleomorphic morphology and more mitotic activity than classic lobular carcinoma. The alveolar variant has classic lobular carcinoma cells that are arranged in globular aggregates of at least 20 cells. The pleomorphic lobular carcinoma exhibits significant cytologic atypia but retains the classic lobular carcinoma pattern of single cell files. The presence of tubules in association with these features defines tubulolobular carcinoma. The signet-ring variant is extremely rare, and the term is reserved for cases where signet-ring cells comprise the exclusive or predominant cell component. The mixed type consists of a mixture of classic and one or more of these variants [1, 8].

We present a case of classic lobular carcinoma but with the rare characteristic of extracellular mucin lakes. This feature is rare but has been documented previously in the literature [2–7].

The differential diagnoses include pure mucinous carcinoma, mixed mucinous-ductal carcinoma, mucinous carcinoma with neuroendocrine differentiation, mixed lobular and ductal carcinoma, solid papillary carcinoma, and mucocele-like tumor. All these tumors have a ductal phenotype and therefore they express E-cadherin [6, 7].

The presence of a bland cytology and discohesive growth is a sign in favor of a lobular carcinoma and E-cadherin immunohistochemical stain should be performed. In fact, the complete loss of membranous E-cadherin confirms the diagnosis of a lobular carcinoma. some nonlobular carcinomas may show decreased expression of E-cadherin; even some subtypes of lobular carcinoma may show an aberrant expression of E-cadherin. However, in nonlobular carcinomas with reduced or insufficient E-cadherin expression, the integrity of the E-cadherin-membrane complex appears to be maintained as evidenced by the membranous expression of *β*-catenin and p120. On the other hand, the aberrant expression of E-cadherin observed in lobular carcinomas is associated with the loss of membranous staining for other molecules of the E-cadherin-membrane complex. Therefore, a study of E-cadherin, *β*-catenin, and p120-catenin may help in the absence of the characteristic morphological features of ILC [7].

The majority of invasive lobular carcinomas (ILCs) are estrogen or progesterone receptor positive and typically do not express HER2 [9].

In our case, ER was positive and PR expression was negative; HER2 expression was absent. Nevertheless, in the case reported by Yu et al. and in one case of the series reported by Cserni et al., HER2 overexpression was shown [5, 7].

The limited number of cases of this rare entity makes it difficult to interpret its clinical behavior and its molecular aspects.

## 4. Conclusion

In summary, the current study describes a rare histologic variant of lobular carcinoma. To the authors' knowledge, only 13 previous cases have been reported. Thus, extracellular mucin production is not an exclusive feature of ductal phenotype. It is prudent to perform E-cadherin immunostain when characteristic histological features of ILC are present for confirmation of diagnosis. This is crucial in order to give the patient the best and earliest treatment.
